# An Uncommon Case of Adolescent Ovarian Teratoma Incarcerated in Inguinal Hernia

**DOI:** 10.1155/2016/4534053

**Published:** 2016-07-20

**Authors:** Marco Pensabene, Vincenza Girgenti, Marcello Cimador, Giuseppe Li Voti, Vito Rodolico, Fortunato Siracusa

**Affiliations:** ^1^Pediatric Surgery, Department of Health Promotion & Mother and Child Care, University of Palermo, Via Alfonso Giordano 3, 90127 Palermo, Italy; ^2^Human Pathology, Department of Health Promotion & Mother and Child Care, University of Palermo, Via Alfonso Giordano 3, 90127 Palermo, Italy

## Abstract

Mature cystic teratoma is the most frequent benign ovarian neoplastic lesion in adolescents and is generally composed of fully differentiated tissue arising from multipotential three germinal layers. It accounts for approximately 50% of benign ovarian tumors in childhood. Rarely, a bilateral, synchronous, or metachronous presentation can be observed, supporting a conservative approach as the first surgical approach. We report a case of an ovarian mature cystic incarcerated in indirect inguinal hernia in a 15-year-old girl undergoing ovary-sparing surgery. To our knowledge this is the first case of such lesion incarcerated in an inguinal hernia reported in literature.

## 1. Introduction

Mature cystic teratoma (MCT), the most common benign ovarian neoplastic lesion in adolescents, arises from the totipotential primitive germ cells [1]. MCT accounts for approximately 70% of benign ovarian neoplasms in women under 30 years of age and 50% of pediatric tumors [2].

Histologically, MCT is characterized by external lining composed of squamous epithelium with an underlying fibroconnective tissue containing hair follicles, sebaceous, eccrine, and apocrine glands. The presence of all the skin appendages in these cysts distinguishes them from epidermoid and sebaceous cysts.

MCT occur most commonly in the ovary, but cases of extragonadal origin have also been described, especially in the omentum, the most common extragonadal site of these parasitic MCT [3].

We report an undescribed case of 15-year-old girl with an ovarian MCT incarcerated in an indirect inguinal hernia, treated with ovary-sparing approach. A dermoid cystic lesion, probably the result of an ovarian autoamputation due to ovarian torsion, was previously described in an indirect inguinal hernia that occurred in a 66-year-old woman [4]. Other reports describe inguinal dermoid cyst [5] and epidermoid inclusion cyst [6].

Although classic treatment of ovarian teratomas was oophorectomy, “ovary-sparing tumor surgery” has become a popular procedure in order to avoid fertility impairment secondary to decreased ovarian tissue reserve and remarkable possibility of contralateral metachronous teratoma development.

## 2. Case Presentation

A 15-year-old female presented at our institution with a slowly growing mass in left groin, noted for 2 years, in menarcheal period. On abdominal examination, the lesion appeared as a smooth, nontender, irreducible mass measuring about 6 × 2,5 cm, fixed in the left inguinal region, and had well-defined margins.

The patient reported a previous failed surgical excision in local anesthesia, attempted in an adult surgery department of another hospital; the first attempt failed because of the unexpected intraoperative detection of the left ovary in inguinal region.

**Figure 1:**
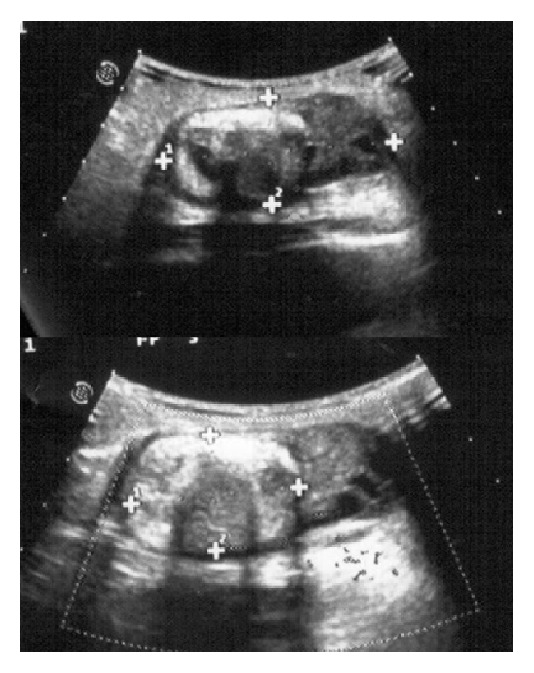
Preoperative US appearance of the inguinal mass, showing the herniated ovary and the teratoma.

The patient was, then, admitted to our institution 2 months later; the first surgical approach and a further US described a multicystic left ovary (5,5 × 2,5 cm) in inguinal region with a hyperechogenous 3,3 cm nodule containing calcific inclusions (Figure 1).

**Figure 2:**
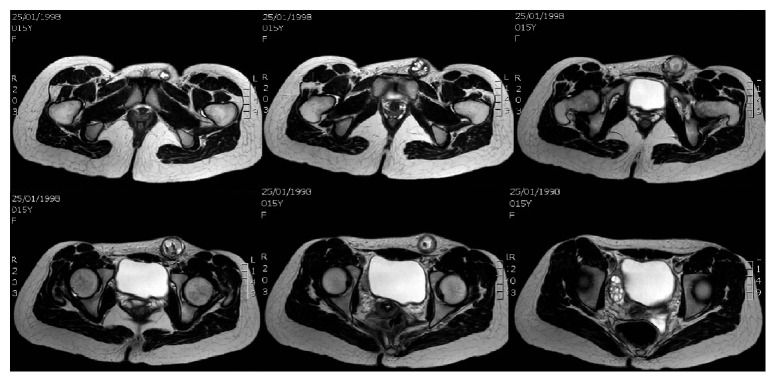
MRI study revealing the ovarian mass herniated in the inguinal region, transverse section.

**Figure 3:**
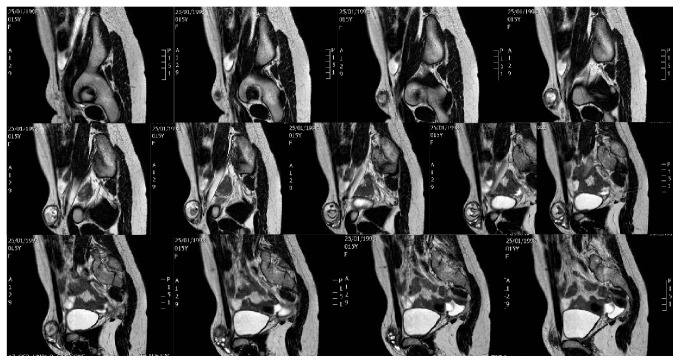
MRI study revealing the ovarian mass herniated in the inguinal region, sagittal section.

Magnetic resonance imaging (MRI) showed a hernia sac extending into the left inguinal region and containing ovarian tissue with an inner cystic-solid mass; there were fat-fluid levels within the mass and a dissection plane between the mass and ovary was evident on MRI (Figures 2 and 3).

Because of the suspected diagnosis of ovarian MCT incarcerated in an inguinal hernia, serum levels of alpha-fetoprotein (AFP), beta-human chorionic gonadotropin (BHCG), and lactate-dehydrogenase (LDH) were investigated, revealing normal serum values.

Surgical excision of the mass and reconstruction of the inguinal region were performed.

**Figure 4:**
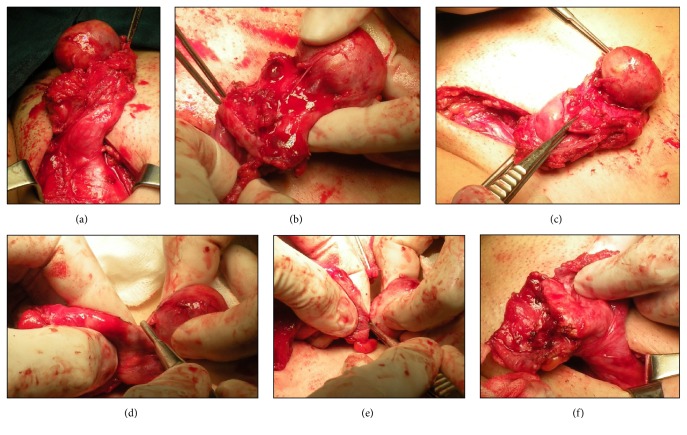
*Intraoperative images*. ((a)–(e)) Excision of the mass; (f) the mass is finally removed and the ovary-sparing surgery is performed.

During surgery the mass was detected at external inguinal ring and isolated from ovarian tissue (Figure 4); the ovary, opened along its major axis, showed evidences of previous surgical operation; nevertheless, it appeared in good macroscopic condition of viability. Fallopian tube elements were isolated and mass was excised radically from ovarian tissue at which margins were sutured. Gonad and adnexa were replaced into abdominal cavity. Finally the hernia defect was repaired through a med mesh plug fixed on the margins of the internal inguinal ring. The inguinal canal was closed in double layer.

The postoperative period was uneventful. Drain was removed on the third postoperative day and the patient was discharged on the third day postoperatively.

At macroscopic pathology, the cystic mass revealed a sebum-like material containing hair. Histopathologic examination of the cyst wall showed keratin composed flooring epithelium with underlying connective tissue including skin adnexa and cylindrical ciliated epithelium. These morphological features were compatible with a MCT.

**Figure 5:**
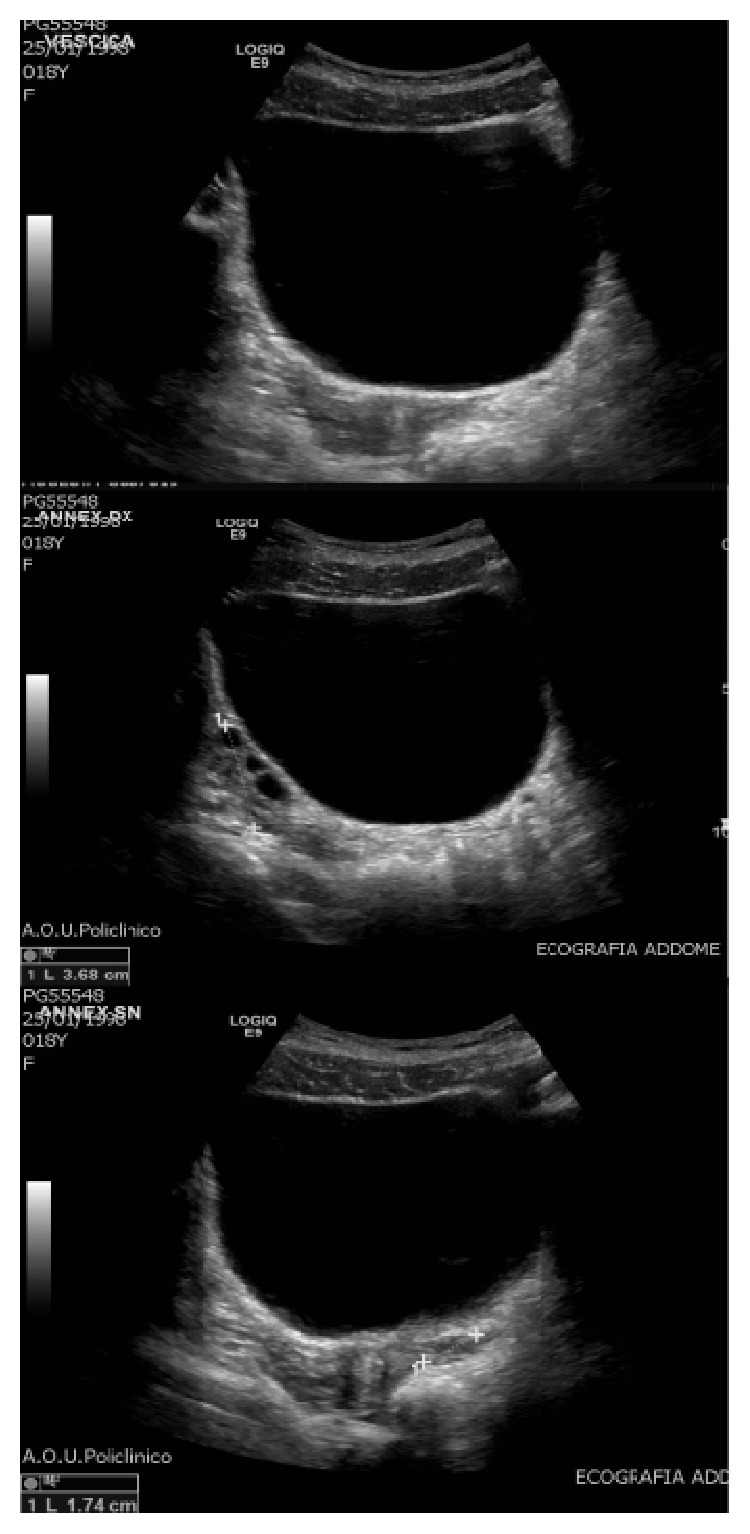
The US examination after 24-month follow-up reveals no recurrence in the left ovary nor metachronous mass in the right ovary.

So, the girl was enrolled in a clinical and imaging follow-up in order to detect any eventual relapse or involvement of the residual ovarian tissue. Pelvic US performed 24 months after surgery showed normal ovaries. Actually, she remains clinically well after a 30-month follow-up (Figure 5).

## 3. Discussion

MCT occur most commonly in the ovary, but extragonadal lesions have also been described. There have been three proposed theories concerning the etiology of those extragonadal sites: (1) primary teratomas originating from displaced germ cells; (2) teratomas developing in a supernumerary ovary, and (3) autoamputation of an ovarian teratoma and reimplantation into an extragonadal site [7].

In our case MCT was found within left ovary inside hernia sac in the left inguinal region. Incarceration of the ovary in the inguinal hernia was probably due to the excessive growth of both ovary and mass under pubertal hormonal stimulation. Adhesion between the tumor and the adjacent structures was probably due to subacute torsion.

Although it is unclear which signal allows the growth of these lesions, it has been suggested that increasing estrogen and progesterone levels stimulate the sebaceous component of these tumors, partially explaining the increase in size seen in MCT after puberty and the arrested growth after menopause [8, 9].

A differential diagnosis of masses in the inguinal region and radiological features includes not only hernias, but also gynecologic, vascular lesions such as lymphangiomas and benign and malignant tumors of the various structures within the inguinal canal. Adnexa or hemorrhagic ovarian cyst in an incarcerated inguinal hernia and primary neuroblastoma of the inguinal canal or neuroblastoma metastasis are some of the lesions reported [6].

Appropriate imaging investigations could avoid unexpected intraoperative features, improving the prognosis by identifying potentially life-threatening lesions.

Pelvic US, which allows good evaluations of localization, size, and structure of the lesion, should be the first approach imaging investigation; computed tomography (CT) scan or MRI can be used in selected case. MCT are mostly in cystic-solid composition with calcifications in 40% of cases [10]. Frequently, they manifest as unilocular cystic lesion, with a hyperechogenous nodule protruding into the cyst lumen, the so-called Rokitansky nodule or dermoid plug. This contains hair follicles and often fragments of bone or teeth. The “dermoid mesh” refers to multiple echogenic linear interfaces, representing hair fibers, that might be seen floating within the cyst. A fat-fluid interface is a distinguishing feature of mature teratomas and it is related to the presence of echogenic sebum floating above hypoechoic serous fluid. The “tip of the iceberg” sign refers to the US appearance of the dermoid plug as an echogenic mass in the near-field resulting in posterior shadowing; thus, the visualization of the bulk of the lesion and posterior wall of the cyst is difficult. On CT and MRI investigations, mature teratomas are easily recognized by the pathognomonic presence of intratumoral fat (adipose tissue within the cyst wall or dermoid plug) and sebum within the cyst lumen [11]. In contrast, immature teratomas mainly consist of solid component with fatty foci. Germ cell tumors with malignant component are frequently larger in size, and the distinction between benign and malign lesions is not easy to achieve only with diagnostic imaging [12]. Recently, Vaysse et al. proposed a 7,5 cm cut-off size as an important cut-off value to differentiate preoperatively between benign and malignant neoplasms [10]. According to some authors, the visualization of normal ovarian tissue adjacent to tumor excludes the malignancy [13].

Serum markers are useful in differentiating benign from malignant ovarian lesion [14]. Elevated preoperative levels of BHCG and AFP are nearly diagnostic of malignant ovarian germ cell tumors; however, these might be present in only 50% of cases [12].

The recommended management of MCT is generally surgical excision due to risk of ovarian torsion, spontaneous rupture, and malignancy occurrence. Nevertheless, malignant transformation of a mature teratoma is a very rare complication, with a reported incidence of 0,17–2% [14]. Autoimmune hemolytic anemia and immune-mediated limbic encephalitis are other rare complications reported [15].

Mature teratomas are classically treated with oophorectomy, either in open or laparoscopic approach. However ovary-sparing surgery has gained an increasing success and consent in recent years. MRI investigation should be performed for proper identification of tumor margins in order to indicate and plan ovary-sparing dissection [16].

Since there is a low recurrence rate (0–4%) [16] for ovary-sparing surgery and in consideration of the possible bilateral involvement (synchronous or metachronous), the ovary-sparing approach should be preferred, if radical excision is anyhow obtained. That could increase fertility rate and fertility period in female population.

However, long-term follow-up with clinical evaluation, serum markers, and imaging studies should be performed in order to detect early relapse or contralateral ovarian involvement.
